# New Method for the Monitoring of Antidepressants in Oral Fluid Using Dried Spot Sampling

**DOI:** 10.3390/ph14121284

**Published:** 2021-12-08

**Authors:** Sofia Soares, Tiago Rosado, Mário Barroso, Eugenia Gallardo

**Affiliations:** 1 Centro de Investigação em Ciências da Saúde, Faculdade de Ciências da Saúde, Universidade da Beira Interior (CICS-UBI), 6200-506 Covilhã, Portugal; sofia_soares_26@hotmail.com (S.S.); tiagorosadofful@hotmail.com (T.R.); 2 Laboratório de Fármaco-Toxicologia, Ubimedical, Universidade da Beira Interior, 6200-284 Covilhã, Portugal; 3 Serviço de Química e Toxicologia Forenses, Instituto de Medicina Legal e Ciências Forenses—Delegação do Sul, 1169-201 Lisboa, Portugal; mario.j.barroso@inmlcf.mj.pt

**Keywords:** antidepressants, biological samples, dried saliva spots, GC–MS/MS, drug monitoring

## Abstract

The increase in the consumption of antidepressants is a public health problem worldwide, as these are a class of compounds widely used in the treatment of several illnesses, such as depression and anxiety. This work aimed to develop and optimize a method for the quantification of a number of antidepressants and their metabolites (fluoxetine, venlafaxine, *O*-desmethylvenlafaxine, citalopram, sertraline, and paroxetine) in 100 µL of oral fluid using the dried saliva spots (DSS) sampling approach and gas chromatography coupled with tandem mass spectrometry (GC–MS/MS). The method was validated, presenting linearity within the studied range, with detection and quantification limits ranging between 10 and 100 ng/mL, and coefficients of determination (R^2^) of at least 0.99 for all analytes. Recoveries were between approximately 13 and 46%. The analysis of precision and accuracy presented acceptable coefficients of variation and relative errors, considering the criteria usually accepted in the validation of bioanalytical procedures. The method herein described is the first to be reported using DSS for the extraction of antidepressants, proving to be a sensitive, simple, and fast alternative to conventional techniques, and capable of being routinely applied in clinical and forensic toxicology scenarios.

## 1. Introduction

Depression is considered to be a serious and chronic mental illness characterized by low mood, loss of interest and desire, sleep disorders, fatigue, suicidal behavior, the ability to compromise social and occupational functions, and affecting individuals regardless of their social or economic status [1,2,3,4,5]. The World Health Organization predicted that this disorder would affect individuals of both sexes and of all ages, being considered the second-leading cause of global disease by 2020, and leading consequently to early deaths due to physical health problems and difficulty accessing health services [1,6].

The most common and effective treatment for moderate-to-severe depression is the administration of antidepressants, which have been increasingly prescribed in recent decades to treat this disorder, but also for other mental health problems such as anxiety, which has led to several expert warnings [7,8,9]. Currently, second-generation antidepressants are the choice of first-line treatment due to their similar efficacy to classic antidepressants and fewer side effects [10,11]. This medication can be prescribed along with other classes of compounds and can, consequently, lead to drug interactions that can be exacerbated by the uncertainty of the dose to be administered. In addition, antidepressants show inter-individual differences, and their therapeutic windows are narrow; as a result, therapeutic drug monitoring is of great interest and importance for patient compliance and safety [7,12,13]. Monitoring allows for the optimization of treatment with these drugs, adjusting and customizing the dosages for each patient and, thus, minimizing toxicity and side effects, avoiding poisoning, lack of response, or non-adherence to treatment, saving costs through the rational use of drugs and resources, and achieving better quality of life [13,14,15,16]. For this monitoring to be possible—and also because the excessive use and abuse of these drugs is verified, culminating in clinical and forensic cases of accidental or voluntary overdose—it is extremely important that analytical methodologies are developed and made available for the identification of antidepressants and their metabolites in biological fluids [10,16].

One of the steps to take into account in an analytical method is the isolation and concentration of analytes of interest from the biological samples under study; the most used procedures for extracting antidepressants are liquid–liquid extraction (LLE) [6,17], solid-phase extraction (SPE) [6,18,19,20], and some miniaturized techniques—such as solid-phase microextraction (SPME) [6,21,22], microextraction by packed sorbent (MEPS) [6,23,24,25], and dispersive liquid–liquid microextraction (DLLME) [6,26,27,28,29,30,31]. There are also several methods involving gas chromatography (GC) and liquid chromatography (LC) coupled with mass spectrometry (MS) [26,30,31,32,33,34,35,36,37,38] or tandem mass spectrometry (MS/MS) [27,39,40,41,42,43], ultraviolet (UV) [22,28,44,45,46,47,48], fluorescence [25], diode array (DAD) or photodiode array (PDA) [24,37,49,50,51], and flame ionization (FID) [29,52,53,54] detectors; more recently, coupling with time-of-flight mass spectrometry (TOF-MS) [21] or quadrupole time-of-flight mass spectrometry (QTOF) [55] has been reported. However, analyses by GC–MS and GS–MS/MS are still the methods of choice, due to their sensitivity and selectivity, which allow them to obtain low limits of quantification, possess separation power for volatile compounds such as the compounds under study, and are robust and generally available in most laboratories.

Presently, oral fluid is considered an excellent alternative in both the clinical and forensic areas for drug determination in biological samples, presenting advantages such as ease of collection, lower risk of adulteration, and a smaller drug detection window, allowing a better correlation with drug effects [56,57,58]. As a way to overcome the disadvantage of classical extraction methods that apply a larger volume of biological sample, miniaturized techniques such as dried matrix spots have been explored, representing a simple and fast procedure compared to other extraction techniques. Applied to blood samples, the technique of dried blood spots (DBS) has been used to determine antidepressants [40,42]. Both the DBS and the dried saliva spots (DSS) techniques have been used in several areas, such as pharmacology, in clinical pharmacokinetic studies, monitoring of drugs, and in the determination of several drugs [59,60,61]. Our group has extensive experience in this area of research, and has already published several papers on methods for the quantification of pharmaceutical compounds based on the DSS sampling approach. Using only 50 µL of oral fluid, Carvalho et al. [62] have determined antiepileptic drugs, Ribeiro et al. [63] have determined methadone and its main metabolite EDDP, and Caramelo et al. [64] have determined antipsychotic drugs. These techniques apply a smaller volume of biological sample and have lower costs of storage and transport compared to classical sampling techniques [60,61], and the DSS, by applying an alternative specimen, becomes an excellent alternative in situations where the amount of sample is limited, as in the case of oral fluid [57,65].

This article reports a methodology for the identification of some of the most frequently prescribed antidepressants, such as fluoxetine (FLX), norfluoxetine (NFLX), citalopram (CIT), sertraline (SRT) and paroxetine (PXT)—which are selective serotonin reuptake inhibitors—and venlafaxine (VLX) and *O-*desmethylvenlafaxine (DVLX) as an antidepressant and metabolite-selective serotonin–norepinephrine reuptake inhibitor, respectively, within the limits of their therapeutic range in only 100 µL of oral fluid samples, using DSS as an extraction procedure and GC–MS/MS analysis. To the best of our knowledge, this is the first application of DSS as an extraction technique to identify these drugs in oral fluid samples, which can be considered as an alternative to the classical techniques normally used in routine laboratory analysis.

## 2. Results and Discussion

### 2.1. Cross-Contribution Evaluation

In the development of the chromatographic method, an important parameter to be evaluated is the cross-contribution of quantifying transitions of each antidepressant under study to the remaining compounds. For this evaluation, the pure and derivatized standards of each antidepressant were injected individually at a concentration of 20 µg/mL, and then analyzed by extracting the chromatograms of the transitions of the remaining analytes in MRM mode (not injected).

The cross-contribution for all compounds was calculated according to the following formula:

(1) $$\mathrm{Contribution}\;(\%)=\frac{\text{absolute peak area of the non-injected antidepressant quantifying transition}}{\text{absolute peak area of the injected antidepressant quantifying transition}}\times 100$$

The results obtained for the cross-contribution evaluation are presented in Table S1 (Supplementary Materials). Because the validation of the analytical method could be compromised for great cross-contributions, for this study it was considered that the contribution would be significant when greater than 5% [66], for which, when present in the same sample, the compounds may present larger areas than those observed when analyzed alone, and which would result in the presence of peaks in the retention times of non-injected analytes in the extraction of their transitions in MRM mode.

As can be seen from the analysis of Table 1, it is possible to conclude that no cross-contribution was observed between the studied antidepressants; consequently, method validation will not be impaired if these drugs are present in the same solution. Thus, it was necessary to use only a mixture of these antidepressants, without changing any of the characteristic transitions of each compound. It was also possible to conclude that there were also no contributions when evaluating the qualifying transitions of the analytes. Therefore, if some of these compounds are present in the same biological specimen, it is still possible to provide a quantitative result for all of them in the therapeutic concentration range.

### 2.2. Optimization of the Extraction Procedure

The evaluation and optimization of the extraction process were performed with samples spiked at 1 μg/mL, starting with the proper selection of the extraction solvent, which should be able to solubilize the analytes of interest, minimizing the co-extraction of other matrix components that can interfere with the chromatographic analysis; in addition, it must be compatible with the analytical technique, and its volatility and polarity must be taken into account. For this univariate study, several solvents were evaluated in triplicate (*n* = 3), in order to choose the one that could obtain the best recoveries of the target analytes. The chosen solvents were methanol, acidified methanol (pH 5), acetonitrile, acidified acetonitrile (pH 5), methanol:acetonitrile (50:50, *v*/*v*), isopropanol, ethyl acetate, hexane, and dichloromethane. These solvents were chosen based on existing scientific literature on the topic [62,63,64,67]. In addition to using methanol and acetonitrile, and taking into account the neutral pH of oral fluid, these solvents were also tested at pH 5 in order to understand whether using these acidified solvents would result in a better extraction yield of the analytes of interest from the DSS cards. The use of this pH is related to the pKa of the compounds.

**Figure 1:**
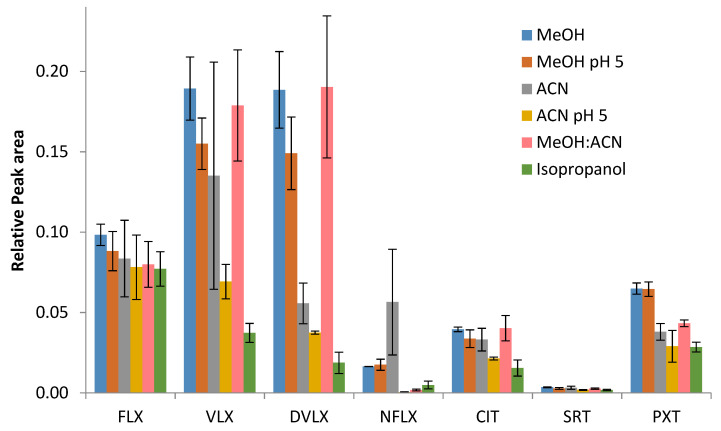
Effects of the different organic solvents and/or mixtures in the extraction process (*n* = 3).

For this first assay, a solvent volume of 2 mL was added to all samples, and the remaining conditions were kept constant, with a 15 min agitation time, overnight drying time, and 5 min of centrifugation at 3500 rpm. The results obtained are shown in Figure 1 and Table S2 (Supplementary Materials). The last three solvents mentioned above were excluded because they yielded the worst chromatographic results and their evaporation time was longer. For the remaining solvents, and after analyzing the results and performing the statistical analysis, it was observed that, in general, methanol seemed to be the solvent with the best extraction recovery, and for which there were no significant differences in relation to the methanol:acetonitrile mixture for any of the compounds, which appeared to be the second best choice for some of the analytes. However, for VLX, DVLX, CIT, SRT, and PXT, there were significant differences between methanol and isopropanol, as well as between methanol and acidified acetonitrile (pH 5), with Friedman’s statistics *p* = 0.005 and *p* = 0.029, respectively, for VLX and CIT; *p* = 0.002 and *p* = 0.016, respectively, for DVLX; *p* = 0.009 and *p* = 0.005, respectively, for SRT; and *p* = 0.009 for the two groups of PXT. The extractions were found to be more efficient when the compounds were not ionized; therefore, methanol was chosen as the extraction solvent. Furthermore, lower standard deviations and associated errors were also observed when methanol was used.

Extraction time, volume of solvent, and drying time were evaluated using the statistical tool Design of Experiments (MINITAB, version 17). The results obtained are shown in the main effects diagrams of Figure S1 (Supplementary Materials). Extraction time showed to generally have little effect, as can be seen in the third column of the main effects graphs; therefore, it was decided to implement a 5 min extraction in order to take full advantage of the speed of this extraction process. On the other hand, the factor extraction volume and sample drying time proved to be the most important conditions in the recovery of most compounds and, therefore, a univariate study was carried out to optimize both parameters.

Three extraction solvent volumes were studied—1, 2, and 3 mL—while 1, 6.50, and 12 h were tested for the drying time, keeping all remaining factors constant. The selection of the solvent volume to be studied must take into account the minimum volume capable of extracting the entire spot, the compromise between the solvent volume and its evaporation speed, and the fact that it is a miniaturized technique. Regarding the drying time, the speed of the extraction process must be taken into account, as it is an alternative to classic extraction methods, and the possibility of processing and analyzing the samples on the same day or, at the latest, the day after their arrival at the laboratory, must also be considered.

**Figure 2:**
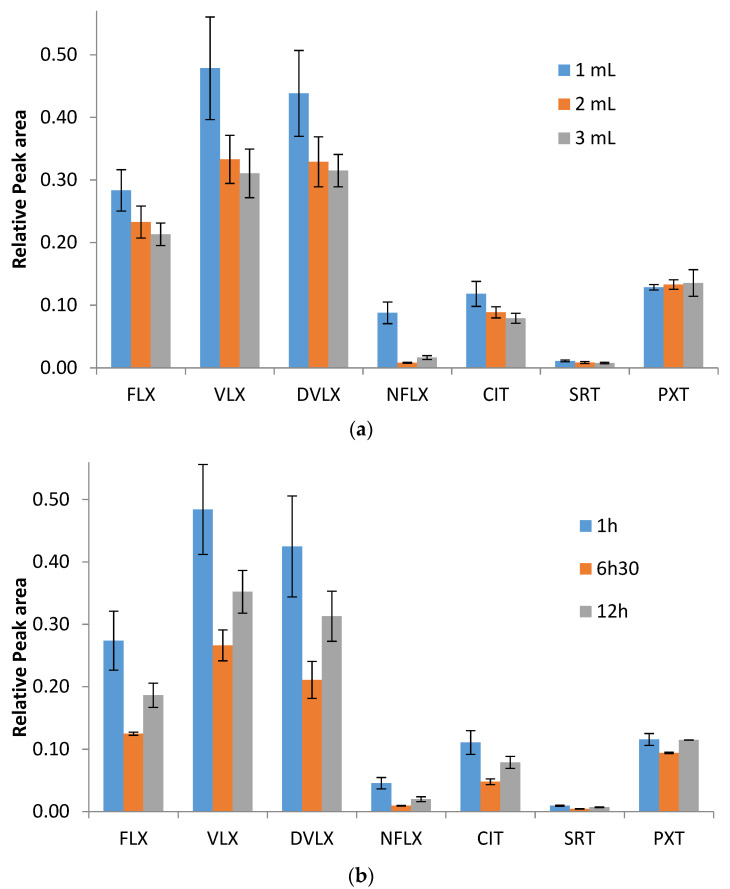
(**a**) Effects of the different solvent volumes (*n* = 3), and (**b**) evaluation of the influence of the drying time of the samples (*n* = 3) in the extraction process.

The obtained results are shown in Figure 2a,b (Supplementary Materials, Tables S3 and S4). Significant differences were observed between 1 and 3 mL for the extraction solvent for CIT, with a Friedman’s statistic of *p* = 0.014; as such, 1 mL of solvent was chosen. With regard to drying time, when comparing the relative areas obtained for 1 and 6.5 h, there was a significant difference, with a Friedman’s statistic of *p* = 0.014, for five of the compounds under study (FLX, VLX, DVLX, CIT, and SRT); therefore, 1 h was selected.

### 2.3. Validation Procedure

The described method was validated according to the accepted international guidelines of the Scientific Working Group for Forensic Toxicology (SWGTOX) [68]. The validation for FLX, VLX, DVLX, CIT, SRT, and PXT was performed following a 3-day validation protocol, and the studied parameters included selectivity; linearity and limits; intra-day, inter-day, and intermediate precision and accuracy; recovery; and stability. NFLX was not included in the validation procedure because it was not possible to achieve linearity; for this reason, this compound was evaluated qualitatively. Therefore, its cross-contribution, extraction process optimization, and recovery were the only studied parameters.

#### 2.3.1. Selectivity

The selectivity of the described method was evaluated by analyzing pools of blank oral fluid samples from 10 different sources, in order to investigate possible interferences in the retention times and selected transitions for the analytes under study.

Identification criteria taken into account for positivity with associated confidence included an absolute retention time within 2% or ±0.1 min of the retention time of the same analyte in the control sample, along with the presence of two transitions per antidepressant. In order to ensure adequate confidence in the identification of these compounds, the maximum allowed tolerances for the relative ionic intensities between the transitions (as a percentage of the base peak) were as follows: if the relative ionic intensity in the control sample was greater than 50%, an absolute tolerance of ±10% was used; if this value was between 25 and 50%, a relative tolerance of ±20% was allowed; if it was between 5 and 25%, an absolute tolerance of ±5% was accepted; and for relative ion intensities of 5% or less, a relative tolerance of ±50% was used [69]. Taking into account these criteria, the analytical method would be considered selective if no compound could be identified in the blank oral fluid samples.

**Figure 3:**
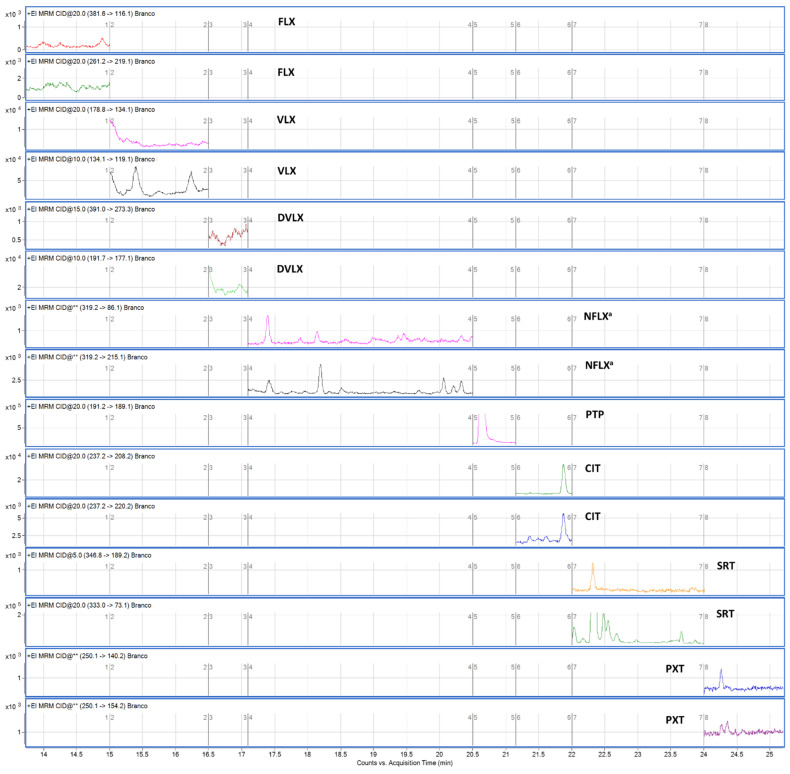
Chromatogram of selected fragments obtained after extraction of a blank oral fluid sample. ^a^: Only for qualitative purposes.

**Figure 4:**
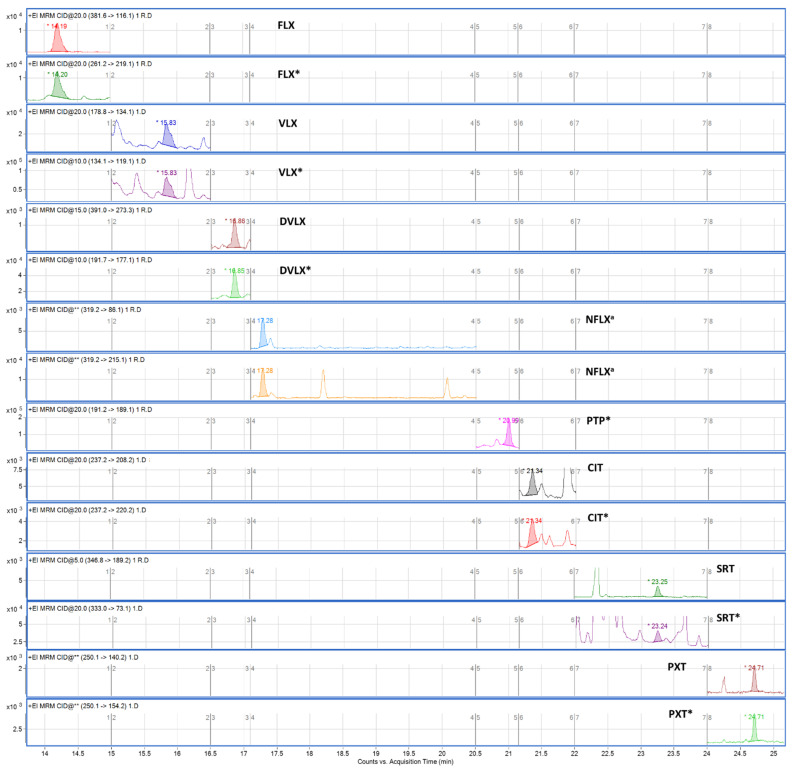
Chromatogram of selected fragments obtained after extraction of the oral fluid samples spiked at the LLOQ. Asterisk indicates quantifier transition. ^a^: Only for qualitative analysis. * Quantitative transition.

After the selectivity assessment, all antidepressants were unequivocally identified in all fortified samples, and no interferences were observed in blank samples, for which they could be detected and/or incorrectly identified as the analyte of interest. Therefore, the method was considered to be selective. Figure 3 and Figure 4 show chromatograms of a blank sample and the sample fortified at the lower limit of quantification (LLOQ), respectively.

#### 2.3.2. Calibration Curves and Limits

Fortified oral fluid samples were processed and analyzed using the extraction procedure described above in the range of 100–500 ng/mL for FLX and VLX, 50–500 ng/mL for DVLX, 20–200 ng/mL for CIT, 40–250 ng/mL for SRT, and 10–100 ng/mL for PXT. The linearity of the method was evaluated using six calibrators with three replicates, and the calibration curves were obtained by plotting the peak area ratio between each compound and the internal standard (IS) against the analyte concentration. The IS protriptyline (PTP) was chosen because it is not commercially available as a therapeutic drug in Portugal; therefore, the chance of it appearing in authentic samples, making quantitative analysis difficult, is very unlikely. In addition, the chemical structure of the IS is similar to that of the studied compounds, which allows for improved linearity, accuracy, and precision, while also minimizing analyte losses during sample preparation.

The acceptance criteria of the calibration curve included a coefficient of determination (R^2^) of at least 0.99, along with accuracy (mean relative error (RE) (bias)) of the calibrators within ±20% of the nominal value [68]. The calibration intervals considered were wide and, to compensate for heteroscedasticity, weighted least squares regressions had to be adopted. The weighting factor 1/× was chosen for all compounds under study. The method was linear within the adopted calibration ranges for all analytes, covering the respective therapeutic ranges, and the calibrators’ RE between the measured and spiked concentrations was within ±20% for all concentrations. With regard to the LLOQ value, this was defined as the lowest concentration that could be measured with adequate precision and accuracy—that is, with a coefficient of variation (CV, %) of less than 20% and an RE (%) within a range of ±20% of the nominal concentration. The limits of detection (LODs) were determined as the lowest concentrations that showed a discrete peak clearly distinguishable from the blank, had a signal-to-noise ratio of at least 3, and corresponded to the LLOQ value for all analytes. The data from the calibration curves and limits are shown in Table 1.

The limits were considered satisfactory taking into account the purpose of the present study to develop a method for quantifying these antidepressants in the context of monitoring—particularly within their respective therapeutic ranges. Some of the published studies include the study by Marasca et al. [23], which developed a methodology to identify some of the antidepressants of this study in oral fluid using volumetric absorptive microsampling (VAMS) as an extraction technique after microsamples were pretreated by means of MEPS, along with analysis by liquid chromatography with sequential spectrophotometric and spectrofluorimetric detection. The authors achieved limit of quantification (LOQ) values of 7 ng/mL for FLX and NFLX, 1 ng/mL for CIT, and 5 ng/mL for SRT, using 100 µL of sample. Shin et al. [20] developed a method for quantifying a wide range of antidepressants—including all of the compounds quantified in this work—in 1 mL oral fluid samples, with extraction by SPE and analysis by LC–MS/MS, achieving an LOQ of 10 ng/mL for all compounds. Applying the same volume of biological specimen, along with the same extraction technique and the same chromatographic analysis, Coulter et al. [8] achieved an LLOQ of 5 ng/mL for FLX and SRT. Additionally, with LC–MS/MS analysis and extraction of 200 µL of oral fluid sample via an automated SPE system, de Castro et al. [70] achieved an LLOQ value of 2 ng/mL for FLX, NFLX, VLX, CIT, SRT, and PXT. However, those methods employed liquid chromatographic–mass spectrometric approaches—a kind of technology not accessible to all laboratories. Nevertheless, our less sensitive instrumentation did not impair the quantification of these antidepressants for the established values. In addition, our method used a smaller volume of biological sample and a smaller volume of organic solvents, in addition to being a much simpler and faster extraction procedure.

#### 2.3.3. Intra-Day, Inter-Day, and Intermediate Precision and Accuracy

Considering the validation criteria, the precision of the method was expressed in terms of CV (%), for which the accepted limit was ≤20% for all concentrations, and the accuracy was characterized in terms of the mean RE (%) between the concentrations measured using the calibration equation and the nominal concentrations, within a ±20% interval.

Regarding intermediate precision and accuracy, quality controls (QCs) were evaluated by analyzing samples at three concentration levels, in triplicate (*n* = 9). CVs typically less than 18% were obtained, with accuracy within a ±14% interval; these results are shown in Table 2.

Inter-day precision and accuracy were evaluated at six concentrations within a 3-day period, for which CVs less than 12% were normally obtained, with an RE value within ±14%; the results are presented in Table 3.

With regard to intra-day precision and accuracy, three concentration levels were evaluated by analyzing six replicates on the same day. The CVs obtained were below 15% at the concentration levels studied, and the mean RE was within the range of ±13% (Table 3).

#### 2.3.4. Extraction Recovery

Regarding the study of absolute recovery, two sets of samples (*n* = 3) were prepared at low, medium, and high concentrations (Supplementary Materials; Table S5). One of the groups, representing 100% recovery, was prepared by spiking the blank oral fluid samples only after extraction, while for the other group the samples were fortified with the analytes under study before the extraction process. The IS was added only after spot extraction for both sets of samples. The recovery results were obtained by comparing the relative peak areas of the analytes from the samples of the second group (obtained via the peak areas of the IS) with those of the analytes from the samples belonging to the first group; the results obtained are shown in Table 4. The extraction efficiencies ranged between approximately 13 and 46% for all compounds which, although low, are acceptable. This may be justified due to an inefficient extraction from the paper, since the recovery of analytes from the spots is related to the efficiency of their extraction process. In order to maximize this recovery, the parameters under study for the optimization of the extraction process were evaluated between considerable intervals, and complementing the univariate study with the analysis by experimental design, in order to achieve the best compromise between the speed of the extraction method and the recovery of analytes. Furthermore, it should be noted that although the recoveries obtained for some of the antidepressants were low, they represent the absolute extraction of the compounds, and did not affect the sensitivity of the method—even when using a low volume of biological sample—since, when using only a volume of 100 μL, small amounts of the analytes under study could be detected and quantified with adequate precision and accuracy.

Since this study represents a new applicability of DSS sampling for antidepressant specimens, the results obtained should be compared to other studies reported in the literature that use other microextraction techniques, or that use the same extraction technique but applied to other biological samples—for instance, blood (DBS). Moretti et al. [40] used DBS sampling followed by SPE to identify a large number of antidepressants, including the analytes of the present study, obtaining recovery values between approximately 32 and 120% for FLX, 87 and 119% for VLX, 85 and 95% for DVLX, and 67 and 99% for CIT, in 85 µL of postmortem blood. The better recoveries compared to the present study can be explained by the differences between biological samples and the better extraction efficiency by the use of SPE after DBS. Marasca et al. [23] developed a methodology to identify antidepressants in oral fluid using VAMS as an extraction technique after microsamples were pretreated by means of MEPS. The authors obtained recovery values between 91 and 96% for FLX, 88 and 91% for NFLX, 91 and 95% for CIT, and 90 and 95% for SRT, using the same volume of 100 µL of oral fluid as in the present work. Similarly to the article by Moretti et al. [40], these authors also obtained higher recovery values compared to the described work, which can also be justified by the use of a pretreatment process of samples via a cleaner microextraction technique than DSS, along with subsequent microsampling by VAMS, which enhances the extraction efficiency. In addition, this method has good sensitivity to therapeutic concentration ranges of the antidepressants under study, and DSS can be considered a powerful technique, resulting in a fast and efficient extraction of target analytes with lower sample and solvent consumption in less time.

#### 2.3.5. Stability

Compound stability was evaluated under different conditions and intervals between processed samples, assessed for short-term and freeze/thaw stability, and was studied at the concentrations of the QCs (*n* = 3), at 125, 275, and 450 ng/mL for FLX and VLX; at 62.5, 137.5, and 450 ng/mL for DVLX; at 25, 55, and 180 ng/mL for CIT; at 50, 110, and 225 ng/mL for SRT; and at 12.5, 27.5, and 90 ng/mL for PXT. The samples submitted to stability studies were compared to freshly prepared samples, analyzed and quantified on the same day and using the same calibration curve, for which the concentrations were compared and the respective CVs and REs calculated. Antidepressants were considered stable if the criteria of CVs below 20% and REs within the range of ±20% were met.

To study the stability in processed samples, previously analyzed extracts were reanalyzed again after being stored at room temperature in the equipment’s autosampler for a period of 24 h. The results obtained allowed us to conclude that none of the studied compounds were stable, because the values of the CV and RE parameters did not meet the aforementioned criteria.

Short-term stability was evaluated with oral fluid samples spiked at the above concentrations, and then left at room temperature for 24 h, after which they were applied to the spots for further extraction. The compounds under study were not stable at the lowest concentration studied for each of them, but they were shown to be stable for the remaining concentration levels, with CVs typically lower than 12% and an accuracy within a range of ±20%.

With regard to freeze/thaw stability, oral fluid samples spiked to the concentrations described above were stored at −20 °C for 24 h. After this time, they were thawed at room temperature and refrozen for another 24 h under the same conditions—a cycle that was repeated twice more before the samples were applied to the spots and analyzed. The antidepressants under study proved to be stable for at least three freeze/thaw cycles, since the CVs obtained were below 12% and the mean RE was within the range of ±20% for all concentration levels.

The data related to stability allow the sample analysis to be performed within a comfortable time window, as the analytes under study are not significantly affected by the storage conditions.

#### 2.3.6. Dilution Integrity

For situations where the analytes of interest are present in concentrations that exceed the upper limit of quantification (ULOQ) of the method, it is necessary to proceed with the dilution of authentic samples.

To assess the integrity of the dilution, three dilution factors (1:2, 1:5, and 1:10) were tested for all analytes under study, allowing the concentrations to fall within the linearity range. Dilutions were made with blank oral fluid, allowing an accurate determination of antidepressants after multiplication by the dilution factor.

The results showed CVs below 20% and RE values within the interval of ±20%. Consequently, even highly concentrated samples could be correctly analyzed after proper dilution.

#### 2.3.7. Method Applicability

**Figure 5:**
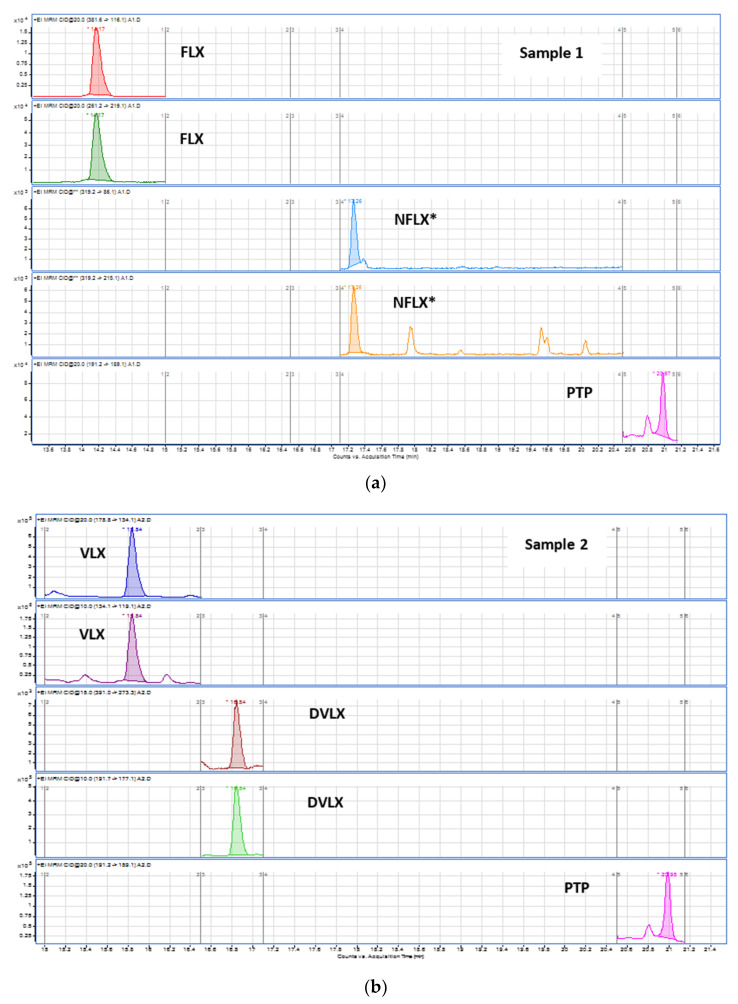

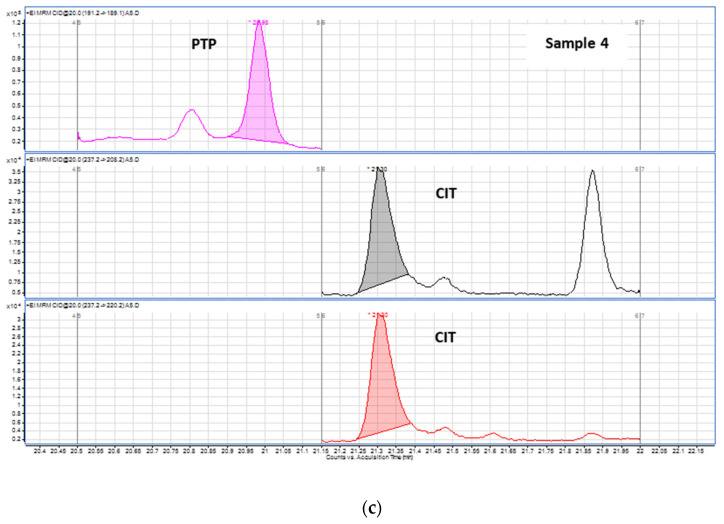
Chromatograms obtained after analysis of authentic oral fluid samples positive for antidepressants: (**a**) FLX consumer (NFLX*—Only for qualitative analysis); (**b**) VLX consumer; (**c**) CIT consumer.

The described and validated procedure was successfully applied in routine analysis of the target antidepressants in authentic oral fluid samples belonging to patients under treatment at the Centro Hospitalar Cova da Beira, in order to demonstrate the applicability of the method. Table 5 shows the results from the analyzed authentic oral fluid samples, and Figure 5 shows the chromatograms obtained when some of the authentic samples were analyzed using the present technique. It can be seen that these oral fluid samples belong to consumers of the compounds under study, with concentration values below but also above the therapeutic range defined for these antidepressants—namely, sample 1, for which a concentration value of 542.1 ng/mL for FLX was obtained; sample 2, with a concentration value of 2033.4 ng/mL for VLX and 701.7 ng/mL for the metabolite DVLX; and sample 4, for which a concentration of 242.8 ng/mL was obtained for CIT. Therefore, the applicability of the method was demonstrated, and it can be used in routine analysis, allowing for the identification and quantification of the antidepressant and its main metabolite whenever present.

## 3. Materials and Methods

### 3.1. Reagents and Standards

Standard solutions of fluoxetine hydrochloride (FLX), venlafaxine hydrochloride (VLX), norfluoxetine (NFLX), citalopram (CIT), and paroxetine (PXT) were provided by Sigma-Aldrich, (St. Louis, MO, USA). *O*-desmethylvenlafaxine (DVLX) was acquired from LGC-Standards (Teddington, London), and sertraline hydrochloride (SRT) was kindly offered by Pfizer (Groton, MA, USA), and their molecular structures and molecular weights are shown in Figure S2 (Supplementary Materials). The internal standard (IS) protriptyline (PTP) was acquired from Sigma-Aldrich (Lisbon, Portugal). Methanol (Merck Co, Darmstadt, Germany) and acetonitrile (Carlo Erba Reagents, Val-de-Reuil, France) were both of analytical grade. Ethyl acetate, 2-propanol, hexane, and dichloromethane were acquired from Fisher Scientific (Loughborough, UK). N-methyl-N-(trimethylsilyl) trifluoroacetamide (MSTFA) and trimethyl chlorosilane (TMCS) were acquired from Macherey-Nagel (Düren, Germany). Whatman™ 903 protein saver cards were acquired from Sigma-Aldrich (Sintra, Portugal).

All standards were acquired at 1 mg/mL. Working standard solutions were prepared by properly diluting the starting solutions with methanol to the final concentrations for the two compound mixtures. Mixture 1 contained FLX, VLX, DVLX, and NFLX at 10 µg/mL, CIT at 4 µg/mL, SRT at 5 µg/mL, and PXT at 2 µg/mL, while mixture 2 contained FLX and VLX at 5 µg/mL, DVLX and NFLX at 2.5 µg/mL, CIT at 1 µg/mL, SRT at 2 µg/mL, and PXT at 0.5 µg/mL. A working solution of the IS was prepared in methanol at a concentration of 1 µg/mL. All of the above solutions were stored in the absence of light at 4 °C.

### 3.2. Biological Specimens

Blank oral fluid samples used in all experiments for the present work were obtained by laboratory staff. Authentic oral fluid samples were analyzed routinely and were obtained from patients under treatment at the Centro Hospitalar Cova da Beira. These samples were sent to our laboratory (Laboratório de Fármaco-Toxicologia, UBIMedical, Covilhã, Portugal) for analysis. All oral fluid specimens were collected by spitting, and without the use of specific collection devices. These samples were stored refrigerated at −20 °C until analysis.

### 3.3. Sample Preparation

The final extraction procedure for the antidepressants was as follows: After homogenization in the vortex mixer, 100 μL of oral fluid was applied to Whatman^®^ 903 protein saver cards and dried for 1 h at room temperature. Then, the spots of each sample were cut with scissors, placed in tubes, and 1 mL of methanol and 20 μL of IS (1 μg/mL) were added, followed by the extraction process—performed with a roller mixer for 5 min at room temperature. The samples were centrifuged for 5 min at 3500 rpm, and the spots were removed from the tubes. The extract was evaporated to dryness under a gentle nitrogen stream, and was subsequently derivatized with 50 μL of MSTFA with 5% TMCS for 2 min in a microwave oven at 800 W. Finally, a 2 μL aliquot of the derivatized extract was injected into the GC–MS/MS system.

The amount of the derivatization agent used is a common parameter in the development of analytical procedures, but also an internal factor optimized by the research group, whereby a compromise is required between the amount used and the chromatographic behavior and signal of the analytes of interest. The derivatizing agent is always added in excess, so as to not be the limiting reagent of the reaction.

### 3.4. Gas Chromatographic and Mass Spectrometric Conditions

Chromatographic analysis was performed using an HP 7890A gas chromatography system equipped with a model 7000B triple-quadrupole mass spectrometer, both from Agilent Technologies (Waldbronn, Germany), along with an MPS2 autosampler and a PTV injector from Gerstel (Mülheim an der Ruhr, Germany). Separation of the antidepressants was achieved using a capillary column (30 m × 0.25 mm I.D., 0.25 μm film thickness) with 5% phenylmethylsiloxane (HP-5MS), provided by J&W Scientific (Folsom, CA, USA).

The initial oven temperature was maintained at 150 °C for 1 min, and then increased to 280 °C at 5 °C/min and held for 4 min, giving a total runtime of 31 min. The injection inlet temperature was set at 250 °C, and the detector temperature was set at 280 °C. The 2 µL of derivatized sample was introduced into the gas chromatograph via splitless injection mode, and the helium was used as a carrier gas with a constant flow rate of 0.8 mL/min. The mass spectrometry was conducted with a filament current of 35 μA and electron energy of 70 eV in the positive electron ionization mode, and nitrogen was utilized as a collision gas at a flow rate of 2.5 mL/min. Data were acquired in the MRM mode using the MassHunter WorkStation Acquisition Software rev. B.02.01 (Agilent Technologies).

The retention time and mass-to-charge ratio (*m/z*) spectra were initially obtained by individually injecting each of the standard antidepressant solutions at a comfortable concentration (100 µg/mL), and then used to identify the different compounds under study. Then, two transitions were chosen for each of the compounds, of which the most abundant transition was used for compound quantitation and the second transition for confirmation purposes. This choice of these transitions was made in order to obtain better selectivity and sensitivity for the analytes and less matrix interference, and the choice of ions for these same transitions was based on the highest masses and most abundant mass peaks (including more specific masses for each compound) in order to maximize the signal-to-noise ratio in the matrix extracts. Table 6 shows the detection criteria—such as retention time, quantifier transition, qualifier transition, and collision energy—selected for each analyte.

## 4. Conclusions

A fully optimized and validated analytical method, which has been shown to be accurate, sensitive, and selective, is described for the simultaneous detection and quantification of five selective serotonin reuptake inhibitor antidepressants (fluoxetine, citalopram, sertraline, and paroxetine) and a selective serotonin–norepinephrine reuptake inhibitor and metabolite (venlafaxine and *O*-desmethylvenlafaxine) in oral fluid samples using DSS and GC–MS/MS. This method was linear within the range of 10–100 ng/mL for all analytes under study, with adequate accuracy and precision, and using only 100 µL of biological sample. The combination of DSS extraction and GC–MS/MS chromatographic analysis proved to be adequate for the determination of these drugs in oral fluid samples. Acceptable recovery values were obtained (13–46%), and good limits of quantification were achieved considering the therapeutic concentration ranges of the studied antidepressants. The low volume of specimen applied and the good sensitivity verified provide significant advantages, especially when there is little specimen availability, which is a problem in the case of the oral fluid, which allows multiple exams to be performed on the same sample. As the first report on the use of DSS as a sampling approach for these compounds, our findings can be considered to provide an alternative to the classical techniques normally implemented, which will result in lower consumption of sample, solvents, and analysis time. Furthermore, the ease of operation allows the routine use of this method in the identification of antidepressants in clinical and forensic toxicology analysis, and its application in authentic biological samples has proven its usefulness in drug monitoring.

## Figures and Tables

**Table 1:** Linearity data.

Analytes	Weight	Linear Range (ng/mL)	Linearity		R^2 a^	LOD/LLOQ (ng/mL)
			Slope ^a^	Intercept ^a^		
FLX	1/×	100–500	0.0022 ± 0.0006	−0.0923 ± 0.0839	0.9965 ± 0.0022	100
VLX	1/×	100–500	0.0079 ± 0.0059	0.1559 ± 0.1499	0.9935 ± 0.0028	100
DVLX	1/×	50–500	0.0083 ± 0.0038	−0.0450 ± 0.0888	0.9954 ± 0.0025	50
CIT	1/×	20–200	0.0021 ± 0.0006	−0.0085 ± 0.0110	0.9935 ± 0.0040	20
SRT	1/×	40–250	0.0003 ± 0.0003	−0.0023 ± 0.0024	0.9964 ± 0.0032	40
PXT	1/×	10–100	0.0041 ± 0.0029	−0.0247 ± 0.0227	0.9959 ± 0.0013	10

^a^: Mean values ± standard deviation.

**Table 2:** Intermediate precision and accuracy (*n* = 9) in oral fluid samples.

Analytes	Spiked (ng/mL)	Measured ^a^ (ng/mL)	CV (%)	RE (%)
FLX	125	133.65 ± 13.57	10.15	6.92
	275	298.07 ± 18.17	6.09	8.39
	450	468.57 ± 58.50	12.48	4.13
VLX	125	126.05 ± 20.47	16.24	0.84
	275	280.38 ± 42.13	15.03	1.96
	450	434.36 ± 51.96	11.96	3.47
DVLX	62.5	66.57 ± 10.17	15.28	6.51
	137.5	141.81 ± 21.86	15.42	3.14
	450	469.84 ± 54.61	11.62	4.41
CIT	25	26.73 ± 3.29	12.30	6.94
	55	49.14 ± 5.87	11.94	−10.65
	180	183.10 ± 27.42	14.97	1.72
SRT	50	49.73 ± 8.61	17.31	−0.54
	110	108.69 ± 13.43	12.35	−1.19
	225	234.13 ± 33.14	14.15	4.06
PXT	12.5	14.18 ± 0.74	5.19	13.43
	27.5	26.42 ± 1.59	6.01	−3.94
	90	100.20 ± 4.33	4.33	11.33

All concentrations in ng/mL; relative error [(measured concentration—spiked concentration/spiked concentration) × 100]. CV: coefficient of variation; RE: relative error. ^a^: Mean values ± standard deviation.

**Table 3:** Inter-day and intra-day precision and accuracy in oral fluid samples.

Analytes	Spiked (ng/mL)	Inter-Day (*n* = 3)		Intra-Day (*n* = 6)	
		CV (%)	RE (%)	CV (%)	RE (%)
FLX	100	1.86	2.44	8.83	7.89
	200	4.19	−0.55	-	-
	250	5.61	−2.34	-	-
	300	2.88	−1.30	4.00	−4.83
	400	1.18	0.35	-	-
	500	0.75	1.40	6.31	−5.39
VLX	100	5.04	0.62	14.64	−0.95
	200	9.63	−1.77	-	-
	250	3.94	1.06	-	-
	300	2.59	−0.38	5.23	−12.99
	400	2.83	0.42	-	-
	500	3.10	−0.22	3.23	−8.39
DVLX	50	7.65	6.34	8.98	−2.52
	100	8.56	−3.73	-	-
	125	11.26	−3.47	-	-
	300	2.79	−1.63	11.63	−12.04
	400	2.24	2.66	-	-
	500	2.01	−0.17	3.35	−3.89
CIT	20	10.27	13.16	13.31	6.10
	40	7.61	−7.00	-	-
	50	8.82	−8.42	-	-
	120	2.88	0.68	8.37	−9.22
	160	1.10	−0.98	-	-
	200	2.14	2.57	5.07	−10.16
SRT	40	3.75	5.23	7.76	1.75
	80	2.57	−6.29	-	-
	100	5.77	−0.70	-	-
	150	1.83	0.83	3.85	−10.15
	200	1.41	−0.14	-	-
	250	1.62	1.07	5.19	−9.56
PXT	10	1.48	12.83	1.44	9.72
	20	1.58	−5.88	-	-
	25	3.78	−9.05	-	-
	60	2.42	−1.04	7.02	−6.55
	80	0.95	1.80	-	-
	100	1.60	1.34	5.57	2.53

All concentrations in ng/mL; relative error [(measured concentration—spiked concentration/spiked concentration) × 100]. CV: coefficient of variation; RE: relative error.

**Table 4:** Recovery of antidepressants (*n* = 3) from the oral fluid samples.

Analytes	Recoveries ^a^ (%)		
	Low-Spiked Concentration	Medium-Spiked Concentration	High-Spiked Concentration
FLX	21.68 ± 0.41	22.70 ± 1.20	23.29 ± 1.28
VLX	46.48 ± 4.44	29.58 ± 3.14	35.79 ± 1.91
DVLX	35.77 ± 2.45	33.88 ± 1.09	38.49 ± 3.21
NFLX	38.30 ± 7.61	13.03 ± 2.38	14.07 ± 2.59
CIT	40.78 ± 6.15	41.57 ± 7.18	35.25 ± 2.04
SRT	24.26 ± 2.70	21.56 ± 2.60	24.42 ± 0.55
PXT	21.53 ± 2.66	21.74 ± 1.91	20.47 ± 0.91

^a^: Mean values ± standard deviation.

**Table 5:** Analysis of the authentic samples.

Samples	Analytes	Concentration (ng/mL)
1	FLX/NFLX	542.1/Detected—Identified
2	VLX/DVLX	2033.4/701.7
3	CIT	32.2
4	CIT	242.8
5	VLX/DVLX	136.0/356.8

**Table 6:** Retention times, selected transitions, and collision energy for the identification of the antidepressants.

Analytes	Retention Time (min)	Quantifier Transition (*m/z*)	Qualifier Transition (*m/z*)	Collision Energy (eV)
FLX	14.20	261.2–219.1	381.6–116.1	20 (20)
VLX	15.86	134.1–119.1	178.8–134.1	10 (20)
DVLX	16.86	191.7–177.1	391.0–273.3	10 (15)
NFLX ^b^	17.29	319.2–215.1	319.2–86.1	5 (15)
PTP ^a^	21.01	191.2–189.1	-	20
CIT	21.33	237.2–208.2	237.2–220.2	20 (20)
SRT	23.27	346.8–189.2	333.0–73.1	5 (20)
PXT	24.72	250.1–154.2	250.1–140.2	20 (15)

^a^: Internal standard; ^b^: only for qualitative effect. The values between brackets in the collision energy (eV) column correspond to the qualifier transition.

## Data Availability

Data is contained within the article.

## Supplementary Materials

The following are available online https://www.mdpi.com/article/10.3390/ph14121284/s1. Table S1: MRM response and cross-contribution of quantifying transitions. Table S2: Effects of the different organic solvents and/or mixtures in the extraction process (n = 3). Figure S1: Main effects plots of drying time, solvent volume and extraction time for the compounds under study. Table S3: Effects of the different solvent volumes (n = 3) in the extraction process. Table S4: Evaluation of the influence of the drying time of the samples (n = 3) in the extraction process. Table S5: Concentrations used in the recovery study. Figure S2: Molecular structures and molecular weights of the target analytes.
