# Supplementary material for: New Method for the Monitoring of Antidepressants in Oral Fluid Using Dried Spot Sampling
Source: Pharmaceuticals (Basel). 2021 Dec 8;14(12):1284. doi: 10.3390/ph14121284 (PMC8709135; doi:10.3390/ph14121284)
Supplement: Supplementary file 1 [file pharmaceuticals-14-01284-s001.zip › pharmaceuticals-1424931-supplementary.pdf]

Supplementary Material

New Method for the Monitoring of Antidepressants in Oral Fluid Using Dried Spot Sampling

Sofia Soares, Tiago Rosado, Mário Barroso and Eugenia Gallardo

Table S1. MRM response and cross-contribution of quantifying transitions.

	**FLX**	**VLX**	**DVLX**	**NFLX[b]**	**PTP[a]**	**CIT**	**SRT**	**PXT**
FLX	100	2.051	0.663	-	-	0.510	-	0.034
VLX	0.176	100	-	-	-	0.084	-	0.005
DVLX	0.078	0.199	100	-	-	0.059	-	-
NFLX[b]	0.030	0.021	-	100	-	0.038	-	0.009
PTP[a]	0.017	0.315	-	-	100	0.037	-	0.003
CIT	0.015	0.011	-	-	0.467	100	-	0.002
SRT	0.016	0.041	-	-	0.051	0.400	100	0.001
PXT	0.018	0.020	-	-	0.022	0.050	0.025	100

[a] Internal standard; [b] Only for qualitative purposes. Underlined antidepressants were injected isolated, and bold antidepressants report the contribution (%) when underlined antidepressants were injected.

Table S2. Effects of the different organic solvents and/or mixtures in the extraction process (n = 3).

	FLX			VLX			DVLX			NFLX			CIT			SRT			PXT		
	**Analyte Area**	**PI Area**	**Relative Area**	**Analyte Area**	**PI Area**	**Relative Area**	**Analyte Area**	**PI Area**	**Relative Area**	**Analyte Area**	**PI Area**	**Relative Area**	**Analyte Area**	**PI Area**	**Relative Area**	**Analyte Area**	**PI Area**	**Relative Area**	**Analyte Area**	**PI Area**	**Relative Area**
	860466	8210737	0.1048	1599875	8210737	0.1949	1713372	8210737	0.2087	135473	8210737	0.0165	327868	8210737	0.0399	31503	8210737	0.0038	499971	8210737	0.0609
**MeOH**	706761	7166032	0.0986	1473541	7166032	0.2056	1395478	7166032	0.1947	117782	7166032	0.0164	292205	7166032	0.0408	24888	7166032	0.0035	483120	7166032	0.0674
	901151	9833349	0.0916	1647105	9833349	0.1675	1595374	9833349	0.1622	146707	8833349	0.0166	373530	9833349	0.0380	32463	9833349	0.0033	652752	9833349	0.0664
Mean	822792.7	8403372.7	0.0984	1573507.0	8403372.7	0.1893	1568074.7	8403372.7	0.1886	133320.7	8070039.3	0.0165	331201.0	8403372.7	0.0396	29618.0	8403372.7	0.0035	545281.0	8403372.7	0.0649
Std Dev	102524.8	1344052.2	0.0066	89736.1	1344052.2	0.0197	160695.6	1344052.2	0.0238	14582.1	842516.1	0.0001	40764.8	1344052.2	0.0014	4124.3	1344052.2	0.0003	93453.2	1344052.2	0.0035
CV	12.46%	15.99%	6.69%	5.70%	15.99%	10.38%	10.25%	15.99%	12.64%	10.94%	10.44%	0.53%	12.31%	15.99%	3.62%	13.93%	15.99%	7.73%	17.14%	15.99%	5.40%
	936354	9526586	0.0983	1472733	9526586	0.1546	1544460	9526586	0.1621	184691	9526586	0.0194	360813	9526586	0.0379	32965	9526586	0.0035	664109	9526586	0.0697
**MeOH pH 5**	761083	8300347	0.0917	1421332	8300347	0.1712	1346030	8300347	0.1622	163027	8300347	0.0196	297592	8300347	0.0359	20298	8300347	0.0024	517922	8300347	0.0624
	872938	11691963	0.0747	1627597	11691963	0.1392	1438157	11691963	0.1230	158149	11691963	0.0135	321991	11691963	0.0275	29930	11691963	0.0026	719602	11691963	0.0615
Mean	856791.7	9839632.0	0.0882	1507220.7	9839632.0	0.1550	1442882.3	9839632.0	0.1491	168622.3	9839632.0	0.0175	326798.7	9839632.0	0.0338	27731.0	9839632.0	0.0028	633877.7	9839632.0	0.0646
Std Dev	88744.1	1717341.9	0.0122	107370.2	1717341.9	0.0160	99299.4	1717341.9	0.0226	14128.0	1717341.9	0.0035	31883.5	1717341.9	0.0055	6613.6	1717341.9	0.0006	104183.3	1717341.9	0.0045
CV	10.36%	17.45%	13.82%	7.12%	17.45%	10.33%	6.88%	17.45%	15.16%	8.38%	17.45%	19.75%	9.76%	17.45%	16.23%	23.85%	17.45%	19.70%	16.44%	17.45%	6.95%
	820705	11426017	0.0718	2460078	11426017	0.2153	469729	11426017	0.0411	379748	11426017	0.0332	285973	11426017	0.0250	26471	11426017	0.0023	423049	11426017	0.0370
**ACN**	566636	8342436	0.0679	904704	8342436	0.1084	515677	8342436	0.0618	470380	8342436	0.0564	306260	8342436	0.0367	25337	8342436	0.0030	278306	8342436	0.0334
	601916	5422523	0.1110	443181	5422523	0.0817	347623	5422523	0.0641	432425	5422523	0.0797	204559	5422523	0.0377	23397	5422523	0.0043	236536	5422523	0.0436
Mean	663085.7	8396992.0	0.0836	1269321.0	8396992.0	0.1352	444343.0	8396992.0	0.0557	427517.7	8396992.0	0.0565	265597.3	8396992.0	0.0332	25068.3	8396992.0	0.0032	312630.3	8396992.0	0.0380
Std Dev	137637.4	3002118.8	0.0238	1056729.6	3002118.8	0.0707	86855.5	3002118.8	0.0127	45514.8	3002118.8	0.0233	53825.2	3002118.8	0.0071	1554.5	3002118.8	0.0010	97879.5	3002118.8	0.0052
CV	20.76%	35.75%	28.50%	83.25%	35.75%	52.29%	19.55%	35.75%	22.75%	10.65%	35.75%	41.19%	20.27%	35.75%	21.28%	6.20%	35.75%	31.40%	31.31%	35.75%	13.68%
	455040	7695163	0.0591	481095	7695163	0.0625	290744	7695163	0.0378	1261	7695163	0.0002	164236	7695163	0.0213	12180	7695163	0.0016	168592	7695163	0.0219
**ACN pH 5**	871928	8797119	0.0991	558904	8797119	0.0635	320134	8797119	0.0364	3271	8797119	0.0004	196804	8797119	0.0224	18462	8797119	0.0021	354876	8797119	0.0403
	632927	8288731	0.0764	676254	8288731	0.0816	317857	8288731	0.0383	4977	8288731	0.0006	170375	8288731	0.0206	16127	8288731	0.0019	205249	8288731	0.0248
Mean	653298.3	8260337.7	0.0782	572084.3	8260337.7	0.0692	309578.3	8260337.7	0.0375	3169.7	8260337.7	0.0004	177138.3	8260337.7	0.0214	15589.7	8260337.7	0.0019	242905.7	8260337.7	0.0290
Std Dev	209189.3	551526.4	0.0201	98244.8	551526.4	0.0107	16350.7	551526.4	0.0010	1860.1	551526.4	0.0002	17305.4	551526.4	0.0009	3175.3	551526.4	0.0003	98686.1	551526.4	0.0099
CV	32.02%	6.68%	25.64%	17.17%	6.68%	15.50%	5.28%	6.68%	2.69%	58.68%	6.68%	57.66%	9.77%	6.68%	4.25%	20.37%	6.68%	14.12%	40.63%	6.68%	34.20%
	415073	5581492	0.0744	1075264	5581492	0.1926	1162117	5581492	0.2082	11650	5581492	0.0021	252786	5581492	0.0453	14181	5581492	0.0025	236608	5581492	0.0424
**MeOH:ACN**	591323	8515466	0.0694	1187896	8515466	0.1395	1192243	8515466	0.1400	10351	8515466	0.0012	265382	8515466	0.0312	20092	8515466	0.0024	357181	8515466	0.0419
	458414	4767391	0.0962	974535	4767391	0.2044	1061614	4767391	0.2227	10421	4767391	0.0022	211456	4767391	0.0444	14932	4767391	0.0031	217876	4767391	0.0457
Mean	488270.0	6288116.3	0.0800	1079231.7	6288116.3	0.1789	1138658.0	6288116.3	0.1903	10807.3	6288116.3	0.0018	243208.0	6288116.3	0.0403	16401.7	6288116.3	0.0027	270555.0	6288116.3	0.0433
Std Dev	91839.8	1971422.1	0.0142	106735.8	1971422.1	0.0346	68401.2	1971422.1	0.0442	730.6	1971422.1	0.0005	28210.1	1971422.1	0.0079	3217.9	1971422.1	0.0004	75602.7	1971422.1	0.0021
CV	18.81%	31.35%	17.77%	9.89%	31.35%	19.34%	6.01%	31.35%	23.20%	6.76%	31.35%	29.19%	11.60%	31.35%	19.62%	19.62%	31.35%	15.09%	27.94%	31.35%	4.73%
	716865	10055622	0.0713	366524	10055622	0.0364	145598	10055622	0.0145	73184	10055622	0.0073	131517	10055622	0.0131	17770	10055622	0.0018	263464	10055622	0.0262
**Isopropanol**	546148	6099096	0.0895	266159	6099096	0.0436	160999	6099096	0.0264	14794	6099096	0.0024	129439	6099096	0.0212	14035	6099096	0.0023	194561	6099096	0.0319
	665028	9409039	0.0707	300355	9409039	0.0319	144594	9409039	0.0154	46815	9409039	0.0050	113989	9409039	0.0121	13295	9409039	0.0014	258444	9409039	0.0275
Mean	642680.3	8521252.3	0.0772	311012.7	8521252.3	0.0373	150397.0	8521252.3	0.0187	44931.0	8521252.3	0.0049	124981.7	8521252.3	0.0155	15033.3	8521252.3	0.0018	238823.0	8521252.3	0.0285
Std Dev	87525.1	2122415.7	0.0107	51024.2	2122415.7	0.0059	9195.3	2122415.7	0.0066	29240.6	2122415.7	0.0024	9576.5	2122415.7	0.0050	2398.7	2122415.7	0.0004	38414.1	2122415.7	0.0030
CV	13.62%	24.91%	13.89%	16.41%	24.91%	15.83%	6.11%	24.91%	35.41%	65.08%	24.91%	49.61%	7.66%	24.91%	32.34%	15.96%	24.91%	24.47%	16.08%	24.91%	10.49%

Mean values ± standard deviation and coefficient of variation.

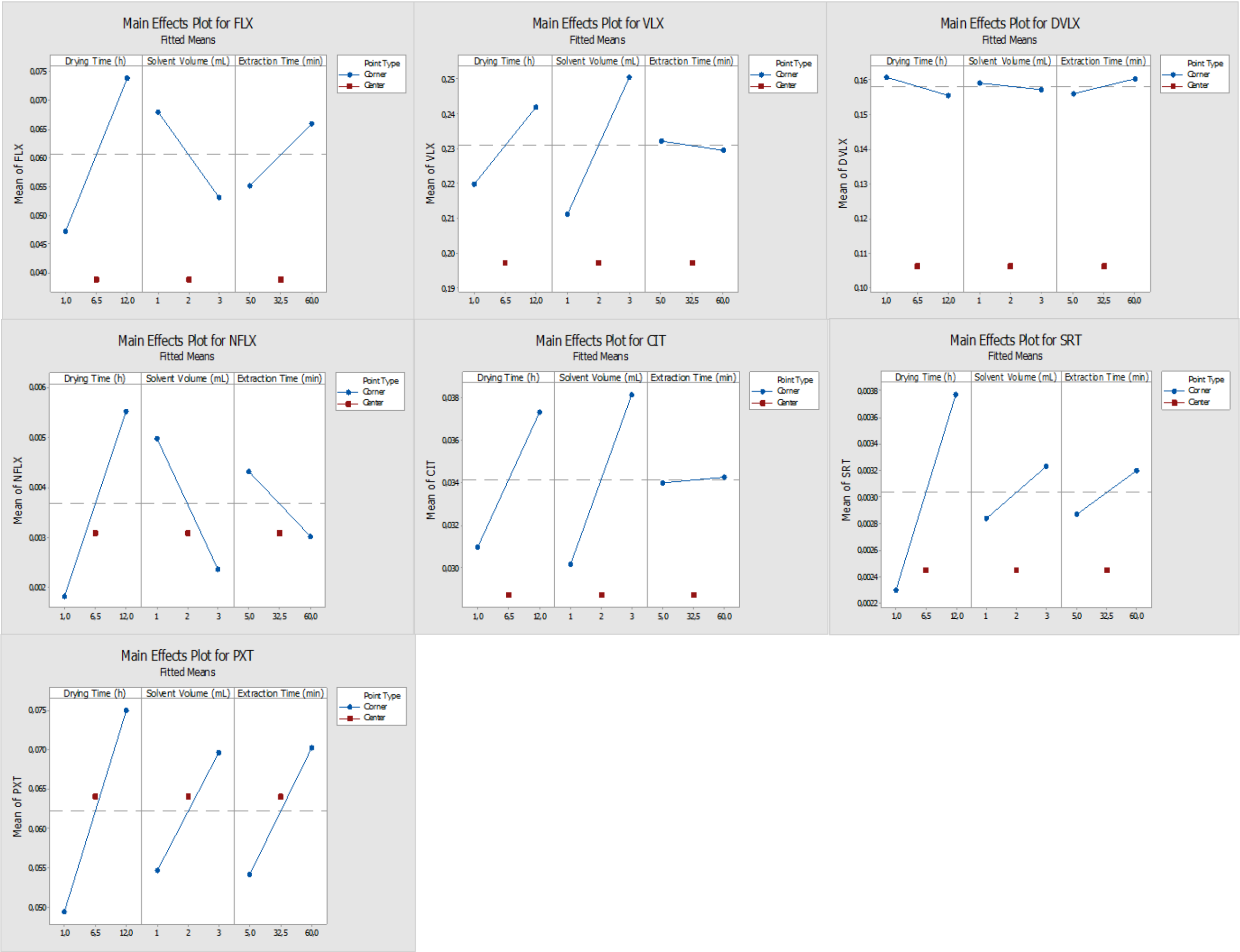

Figure S1. Main effects plots of drying time, solvent volume and extraction time for the compounds under study.

Table S3. Effects of the different solvent volumes (n = 3) in the extraction process.

	**FLX**			**VLX**			**DVLX**			**NFLX**			**CIT**			**SRT**			**PXT**		
	**Analyte Area**	**PI Area**	**Relative Area**	**Analyte Area**	**PI Area**	**Relative Area**	**Analyte Area**	**PI Area**	**Relative Area**	**Analyte Area**	**PI Area**	**Relative Area**	**Analyte Area**	**PI Area**	**Relative Area**	**Analyte Area**	**PI Area**	**Relative Area**	**Analyte Area**	**PI Area**	**Relative Area**
**1 mL**	1398967	4932691	0.2836	2257212	4932691	0.4576	2088925	4932691	0.4235	372752	4932691	0.0756	586794	4932691	0.1190	62242	4932691	0.0126	618794	4932691	0.1254
	1441689	5763604	0.2501	2357924	5763604	0.4091	2181788	5763604	0.3785	576644	5763604	0.1000	562513	5763604	0.0976	61247	5763604	0.0106	834573	5763604	0.1448
	561887	1776521	0.3163	1010430	1776521	0.5688	911573	1776521	0.5131	155794	1776521	0.0877	244221	1776521	0.1375	17589	1776521	0.0099	233886	1776521	0.1317
Mean	1134181.0	4157605.3	0.2833	1875188.7	4157605.3	0.4785	1727428.7	4157605.3	0.4384	368396.7	4157605.3	0.0878	464509.3	4157605.3	0.1180	47026.0	4157605.3	0.0110	426340.0	3354606.0	0.1286
Std Dev	496081.3	2103515.2	0.0331	750594.0	2103515.2	0.0819	708075.7	2103515.2	0.0685	210458.8	2103515.2	0.0122	191161.2	2103515.2	0.0200	25498.0	2103515.2	0.0014	272171.1	2231749.2	0.0044
CV	43.74%	50.59%	11.67%	40.03%	50.59%	17.11%	40.99%	50.59%	15.63%	57.13%	50.59%	13.95%	41.15%	50.59%	16.91%	54.22%	50.59%	12.73%	63.84%	66.53%	3.41%
**2 mL**	1355079	5946378	0.2279	1949260	5946378	0.3278	1934413	5946378	0.3253	45920	5946378	0.0077	536028	5946378	0.0901	44180	5946378	0.0074	777486	5946378	0.1307
	1533518	5891277	0.2603	2202014	5891277	0.3738	2182624	5891277	0.3705	49014	5891277	0.0083	568402	5891277	0.0965	61326	5891277	0.0104	853933	5891277	0.1449
	1390695	6618765	0.2101	1968095	6618765	0.2974	1925309	6618765	0.2909	57526	6618765	0.0087	522841	6618765	0.0790	54363	6618765	0.0082	879523	6618765	0.1329
Mean	1426430.7	6152140.0	0.2328	2039789.7	6152140.0	0.3330	2014115.3	6152140.0	0.3289	50820.0	6152140.0	0.0082	542423.7	6152140.0	0.0885	53289.7	6152140.0	0.0087	879523.0	6618765.0	0.1329
Std Dev	94434.6	405047.2	0.0254	140805.7	405047.2	0.0385	146003.8	405047.2	0.0399	6010.1	405047.2	0.0005	23444.2	405047.2	0.0089	8623.2	405047.2	0.0015	53088.8	405047.2	0.0077
CV	6.62%	6.58%	10.93%	6.90%	6.58%	11.55%	7.25%	6.58%	12.14%	11.83%	6.58%	5.93%	4.32%	6.58%	10.00%	16.18%	6.58%	17.79%	6.04%	6.12%	5.76%
**3 mL**	1387067	5959096	0.2328	2037785	5959096	0.3420	2045755	5959096	0.3433	111242	5959096	0.0187	525325	5959096	0.0882	54377	5959096	0.0091	896597	5959096	0.1505
	1175111	5602973	0.2097	1808657	5602973	0.3228	1731170	5602973	0.3090	87572	5302973	0.0165	416847	5602973	0.0744	39227	5602973	0.0070	683302	5602973	0.1220
	1233994	6254875	0.1973	1670446	6254875	0.2671	1831480	6254875	0.2928	89453	6254875	0.0143	464531	6254875	0.0743	44659	6254875	0.0071	752669	6254875	0.1203
Mean	1265390.7	5938981.3	0.2133	1838962.7	5938981.3	0.3106	1869468.3	5938981.3	0.3150	96089.0	5838981.3	0.0165	468901.0	5938981.3	0.0789	46087.7	5938981.3	0.0078	824633.0	6106985.5	0.1354
Std Dev	109410.5	326416.2	0.0180	185535.2	326416.2	0.0389	160696.2	326416.2	0.0258	13156.5	487185.8	0.0022	54370.9	326416.2	0.0080	7675.4	326416.2	0.0012	108797.8	326416.2	0.0213
CV	8.65%	5.50%	8.44%	10.09%	5.50%	12.53%	8.60%	5.50%	8.18%	13.69%	8.34%	13.24%	11.60%	5.50%	10.11%	16.65%	5.50%	15.32%	13.19%	5.34%	15.73%

Mean values ± standard deviation and coefficient of variation.

Table S4. Evaluation of the influence of the drying time of the samples (n = 3) in the extraction process.

	FLX			VLX			DVLX			NFLX			CIT			SRT			PXT		
	**Analyte Area**	**PI Area**	**Relative Area**	**Analyte Area**	**PI Area**	**Relative Area**	**Analyte Area**	**PI Area**	**Relative Area**	**Analyte Area**	**PI Area**	**Relative Area**	**Analyte Area**	**PI Area**	**Relative Area**	**Analyte Area**	**PI Area**	**Relative Area**	**Analyte Area**	**PI Area**	**Relative Area**
	654828	2041526	0.3208	1051699	2041526	0.5152	960109	2041526	0.4703	150272	3291526	0.0457	259581	2041526	0.1272	19237	2041526	0.0094	212379	2041526	0.1040
**1h**	1112530	4921158	0.2261	1977254	4921158	0.4018	1630358	4921158	0.3313	192915	4921158	0.0392	441408	4921158	0.0897	40568	4921158	0.0082	618248	4921158	0.1256
	922869	3362676	0.2744	1801136	3362676	0.5356	1589765	3362676	0.4728	174892	3362676	0.0520	387835	3362676	0.1153	34874	3362676	0.0104	394954	3362676	0.1175
Mean	896742.3	3441786.7	0.2738	1610029.7	3441786.7	0.4842	1393410.7	3441786.7	0.4248	172693.0	3858453.3	0.0456	362941.3	3441786.7	0.1107	31559.7	3441786.7	0.0093	408527.0	3441786.7	0.1157
Std Dev	229966.8	1441445.1	0.0473	491481.7	1441445.1	0.0721	375798.7	1441445.1	0.0810	21406.4	921016.6	0.0064	93434.7	1441445.1	0.0191	11045.0	1441445.1	0.0011	203274.6	1441445.1	0.0095
CV	25.64%	41.88%	17.29%	30.53%	41.88%	14.89%	26.97%	41.88%	19.06%	12.40%	23.87%	14.04%	25.74%	41.88%	17.29%	35.00%	41.88%	11.40%	49.76%	41.88%	8.20%
	818099	6425327	0.1273	1880877	6425327	0.2927	1565901	6425327	0.2437	61637	6425327	0.0096	341922	6425327	0.0532	29308	6425327	0.0046	612225	6425327	0.0953
**6h30**	1020424	8182978	0.1247	2142416	8182978	0.2618	1658741	8182978	0.2027	80810	8182978	0.0099	366379	8182978	0.0448	33851	8182978	0.0041	762445	8182978	0.0932
	1165488	9517734	0.1225	2321198	9517734	0.2439	1771756	9517734	0.1862	84059	9517734	0.0088	433822	9517734	0.0456	38888	9517734	0.0041	895593	9517734	0.0941
Mean	1001337.0	8042013.0	0.1248	2114830.3	8042013.0	0.2661	1665466.0	8042013.0	0.2109	75502.0	8042013.0	0.0094	380707.7	8042013.0	0.0479	34015.7	8042013.0	0.0043	756754.3	8042013.0	0.0942
Std Dev	174479.3	1551015.3	0.0024	221452.9	1551015.3	0.0247	103092.1	1551015.3	0.0296	12116.8	1551015.3	0.0005	47596.1	1551015.3	0.0047	4792.1	1551015.3	0.0003	141769.7	1551015.3	0.0011
CV	17.42%	19.29%	1.95%	10.47%	19.29%	9.28%	6.19%	19.29%	14.05%	16.05%	19.29%	5.72%	12.50%	19.29%	9.73%	14.09%	19.29%	6.13%	18.73%	19.29%	1.12%
	843898	4147471	0.2035	1565663	4147471	0.3775	1480123	4147471	0.3569	83412	4207471	0.0198	373510	4147471	0.0901	30997	4147471	0.0075	475822	4147471	0.1147
**12h**	1082296	5695246	0.1900	2082847	5695246	0.3657	1731406	5695246	0.3040	97408	5695246	0.0171	421608	5695246	0.0740	39176	5695246	0.0069	682954	5695246	0.1199
	1064538	6455256	0.1649	2020334	6455256	0.3130	1793964	6455256	0.2779	146243	6455256	0.0227	469487	6455256	0.0727	40825	6455256	0.0063	741202	6455256	0.1148
Mean	996910.7	5432657.7	0.1861	1889614.7	5432657.7	0.3521	1668497.7	5432657.7	0.3129	109021.0	5452657.7	0.0199	421535.0	5432657.7	0.0789	36999.3	5432657.7	0.0069	633326.0	5432657.7	0.1148
Std Dev	132810.0	1176087.7	0.0196	282286.2	1176087.7	0.0344	166108.8	1176087.7	0.0402	32986.1	1143359.6	0.0028	47988.5	1176087.7	0.0097	5263.2	1176087.7	0.0006	139477.0	1176087.7	0.0001
CV	13.32%	21.65%	10.52%	14.94%	21.65%	9.76%	9.96%	21.65%	12.86%	30.26%	20.97%	13.98%	11.38%	21.65%	12.23%	14.23%	21.65%	8.34%	22.02%	21.65%	0.06%

Mean values ± standard deviation and coefficient of variation

Table S5. Concentrations used in the recovery study.

Compound	Concentrations (ng/mL)		
**FLX**	80	200	400
**VLX**	90	180	300
**DVLX**	120	240	400
**NFLX**	30	60	100
**CIT**	60	120	200
**SRT**	60	120	200
**PXT**	30	60	100

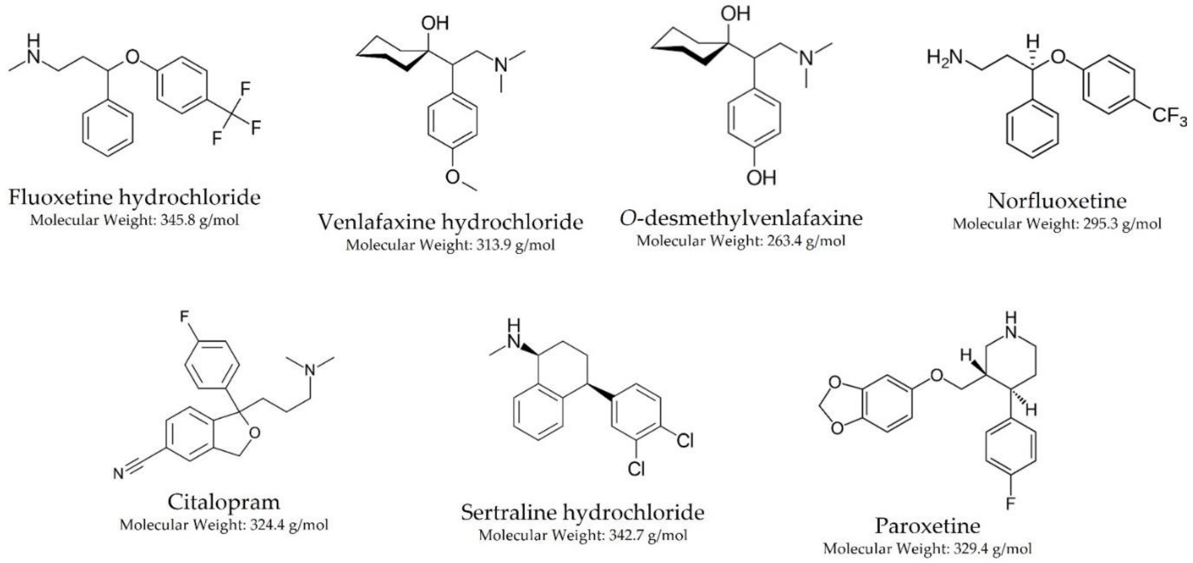

Figure S2. Molecular structures and molecular weights of the target analytes.
